# High Concentrations of Uric Acid and Angiotensin II Act Additively to Produce Endothelial Injury

**DOI:** 10.1155/2020/8387654

**Published:** 2020-05-22

**Authors:** Quan Hong, Liyuan Wang, Zhiyong Huang, Zhe Feng, Shaoyuan Cui, Bo Fu, Guangyan Cai, Xiangmei Chen, Di Wu

**Affiliations:** ^1^Department of Nephrology, Chinese PLA General Hospital, Chinese PLA Institute of Nephrology, State Key Laboratory of Kidney Diseases, National Clinical Research Center of Kidney Diseases, Chinese PLA General Hospital, Beijing 100853, China; ^2^Kidney Department, Halison International Peace Hospital, Hengshui City 053000, China; ^3^Department of Nephrology, The 175th Hospital of PLA, Zhangzhou Fujian 036300, China

## Abstract

Renin angiotensin (Ang) system (RAS) activation in metabolic syndrome (MS) patients is associated with elevated uric acid (UA) levels, resulting in endothelial system dysfunction. Our previous study demonstrated that excessive UA could cause endothelial injury through the aldose reductase (AR) pathway. This study is the first to show that a high concentration of Ang II in human umbilical vein endothelial cells (HUVECs) increases reactive oxygen species (ROS) components, including O_2_^·-^ and H_2_O_2_, and further aggravates endothelial system injury induced by high UA (HUA). In a MS/hyperuricemia model, nitric oxide (NO) production was decreased, followed by a decrease in total antioxidant capacity (TAC), and the concentration of the endothelial injury marker von Willebrand factor (vWF) in the serum was increased. Treatment with catalase and polyethylene glycol covalently linked to superoxide dismutase (PEG-SOD) to individually remove H_2_O_2_ and O_2_^·-^ or treatment with the AR inhibitor epalrestat decreased ROS and H_2_O_2_, increased NO levels and TAC, and reduced vWF release. Taken together, these data indicate that HUA and Ang II act additively to cause endothelial dysfunction via oxidative stress, and specific elimination of O_2_^·-^ and H_2_O_2_ improves endothelial function. We provide theoretical evidence to prevent or delay endothelial injury caused by metabolic diseases.

## 1. Introduction

Due to economic development and an improved standard of living, obesity in adults and children is increasing. Metabolic syndrome (MS) is related to obesity, atherosclerosis, hypertension, disturbance of lipid metabolism, and insulin resistance [1]. Angiotensin (Ang) II is the most well-studied factor related to MS. The mechanism of endothelial injury involves oxidative stress, redox signal pathway activation, and cytokine and inflammatory factor reactivation [2]. MS is associated with hyperuricemia [3–5]. Uric acid (UA) is an antioxidant, and an increase in its levels is protective against endothelial injury [6]. Our previous study found that UA has an antioxidant capacity; however, a high concentration of UA (HUA) also causes endothelial dysfunction [7–12].

The production of reactive oxygen species (ROS), a decrease in endothelial nitric oxide (NO) synthase (eNOS) activity, and a decline in NO release are thought to comprise the prominent mechanism of endothelial injury [13]. Both metabolic and pathological processes can generate ROS, and an attenuated scavenging capacity leads to an imbalance of oxidants and antioxidants, causing oxidative stress. Whether both HUA and Ang II cause endothelial injury via redox signaling pathway activation and prevent NO production is unknown. Some evidence has demonstrated that the aldose reductase (AR) pathway, which is related to ROS production, is activated during endothelial injury [7, 14]. Whether AR activation participates in ROS generation in endothelial injury caused by HUA and Ang II remains unknown.

Therefore, in this study, we used human umbilical vein endothelial cells (HUVECs) and constructed an MS model. An ROS scavenger or AR inhibitor was administered to examine the relationship and the mechanism of endothelial injury caused by hyperuricemia and Ang II.

## 2. Materials and Methods

### 2.1. Animals

Male spontaneously hypertensive (SHR) rats were obtained from Beijing Vital River Laboratory Animal Technology Co., Ltd. (Beijing, China) at approximately 10 weeks of age. The experimental protocol was approved by the Institutional Animal Care and Use Committee of the Chinese PLA General Hospital. All animals were housed simultaneously in individual stainless-steel wire cages with a controlled temperature (22°C to 24°C) and relative humidity (40-50%) and maintained on a reverse 12-hour dark and light cycle.

### 2.2. Diet and Experimental Design

All animals were initially provided with high-fat rat chow for 1 week and were maintained on this diet. Food and water were consumed ad libitum. The rats were then randomly divided into 5 groups of 6 rats. The MS group consumed food ad libitum; the MS with the HUA group was administered oxonic acid (250 mg kg^−1^ d^−1^) and UA (250 mg kg^−1^ d^−1^) via intraperitoneal injection for 10 consecutive days; the catalase group (HUA+catalase) received 12 kU kg^−1^ d^−1^ catalase (Sigma, USA) via intraperitoneal injection; the SOD group (HUA+polyethylene glycol covalently linked to superoxide dismutase (PEG-SOD)) received 2 × 10^4^ U kg^−1^ d^−1^ PEG-SOD (Sigma, USA) via intramuscular injection; and the epalrestat group received 100 mg kg^−1^ d^−1^ epalrestat (Yangtze River Pharmaceutical Group, Jiangsu, China) via irrigation. On the 10th day, animals were administered intraperitoneal anesthesia consisting of pentobarbital sodium (40 mg/kg), and blood, urine, and kidney tissue were collected.

### 2.3. Cell Culture

HUVECs were purchased from YRbio (Cat# NC006, Changsha, China) and cultured in RPMI-1640 media supplemented with 10% fetal bovine serum (FBS) at 37°C in a humidified incubator in a 5% CO_2_ atmosphere. The final concentrations of UA (Sigma, USA) and Ang II (Sigma, USA) in the medium were 600 *μ*mol/L and 1 × 10^−7^ mol/L, respectively. Cells were stimulated by HUA or Ang II for 24 h, and then, the supernatant or protein was harvested for assay. For the NO assay, the medium was replaced with Dulbecco's modified Eagle's medium (DMEM).

### 2.4. Measurement of NO Levels in Culture Supernatants and Serum of Animals

Before treatment with chemicals or UA, the medium was replaced with DMEM. The culture supernatant or serum of animals was centrifuged and subjected to NO level evaluation using the Nitric Oxide Assay Kit (Applygen Technologies, China) according to the manufacturer's instructions. The endpoint compound was NO_2_^−^ [7].

### 2.5. Intracellular ROS Assay

The ROS assay used was referenced in a previous study [7]. Cells were seeded onto 35 mm confocal dishes (with a cover glass) and classified into the control, HUA, Ang II, or HUA+Ang II group. After 24 h, cells were incubated with the total oxidative stress indicator chloromethyl derivative dichlorodihydrofluorescein diacetate (CM-H_2_DCFDA, Beyotime, Nanjing China, 5 *μ*M) for 30 min in the dark at 37°C. After three washes with PBS, green fluorescence was visualized using a laser scanning confocal microscope (FV1000, Olympus, Tokyo, Japan) at an excitation wavelength of 488 nm and an emission wavelength of 515 nm. The detection of intracellular ROS components was performed as follows. For O_2_^·−^, cells were incubated with 4 *μ*mol/L Mito-SOX Red (Invitrogen) in the dark at 37°C for 10 min, and red fluorescence was observed at an excitation wavelength of 510 nm and an emission wavelength of 580 nm. For H_2_O_2_, cells were incubated with 30 *μ*mol/L BES-H_2_O_2_ (Seebio, China) in the dark at 37°C for 1 h, and green fluorescence was observed at an excitation wavelength of 485 nm and an emission wavelength of 515 nm. For ^·^OH, cells were incubated with 100 *μ*mol/L proxyl fluorescamine (Invitrogen) in the dark at 37°C for 30 min, and green fluorescence was observed at an excitation wavelength of 488 nm and an emission wavelength of 520 nm. For ^1^O_2_, cells were incubated with 20 *μ*mol/L trans-1-(2′-methoxyvinyl) pyrene (Invitrogen) in the dark at 37°C for 10 min, and blue fluorescence was observed at an excitation wavelength of 405 nm and an emission wavelength of 460 nm. For ONOO^−^, cells were incubated with 10 *μ*mol/L dihydrorhodamine 123 (Santa Cruz) in the dark at 37°C for 30 min, and green fluorescence was observed at an excitation wavelength of 488 nm and an emission wavelength of 520 nm.

### 2.6. Western Blotting

For Western blotting [7], proteins were extracted from tissues or cells using RIPA lysis buffer (50 mM Tris-HCl, pH 7.5, 150 mM NaCl, 0.5% deoxycholate, 1% Nonidet P-40, 0.1% SDS, 1 mM PMSF, and protease cocktail at 1 *μ*g/mL). Protein concentrations were measured using a BCA kit (Pierce). Protein samples (50 *μ*g per lane) were separated by 12% SDS-PAGE and transferred to nitrocellulose (NC) membranes. Membranes were incubated overnight at 4°C in 5% nonfat milk followed by incubation with primary antibodies, anti-eNOS and p-eNOS^ser1177^ (Santa Cruz Biotechnology, USA) and Nox2 and Nox4 (Cell Signaling Technology, MA, USA); *β*-actin (Abcam, USA) was used as an internal control. Immunoreactive bands were visualized using ECL reagent (Santa Cruz Biotechnology, USA) according to the manufacturer's instructions. Protein band intensities were quantified using Quantity One software (Bio-Rad Laboratories, Hercules, CA, USA).

### 2.7. Measurement of Serum H_2_O_2_, Total Antioxidant Capacity (TAC)

The serum H_2_O_2_ levels of animals were assayed using a hydrogen peroxide assay kit (NJJCbio, Nanjing, China) examining the end product, Mn^2+^. The TAC of animals was assayed using the ferric reducing antioxidant power (FRAP) method with a commercial kit (Beyotime Institute of Biotechnology, Nanjing, China).

### 2.8. Measurement of von Willebrand Factor (vWF) and Endothelin-1 (ET1) Levels

Confluent HUVECs were starved in a serum-free medium supplemented with 1% BSA for 4 h, then incubated with Ang II or HUA for 24 h. The medium was collected, and the remaining cells were lysed to determine total vWF levels. Relative amounts of VWF were determined using a von Willebrand factor ELISA kit (Invitrogen, USA). Basal and stimulated release is presented as a percentage of the total vWF present in the cells. Similarly, the serum vWF level of animals was assayed with the commercial ELISA kit (Wuxi Donglin Sci & Tech Development Co., Ltd., China) and was presented with a quantification of ng/mL. ET1 in a cell medium or serum of animals was measured using the ET1 ELISA kit (Elabscience, China) followed with the instruction.

### 2.9. NADPH Oxidase Activity Assay

In order to assay NADPH oxidase activity, cell was lysed with Hepengbio lysis buffer and preparing the assay protocol followed the instruction provided by Hepengbio Ltd., China. Total NOX activities were calculated and showed with U/*μ*g protein.

### 2.10. Intraperitoneal Glucose Tolerance Test

An intraperitoneal glucose tolerance test (ipGTT) was performed when the animals were 4 months or 1 year old. The animals were injected intraperitoneally with a 30% (*w*/*v*) D-glucose solution (2 g glucose/kg body weight) after an overnight fast. Blood samples were taken from the cut tip of the tail immediately before and 10, 30, 60, and 120 min after glucose administration. Blood glucose concentrations were measured with test reagent strips (ExacTech w; Baxter Travenol, Deerfield, IL, USA). Serum immunoreactive insulin concentrations were measured with radioimmunoassay (Insulin RIA kit; Pharmacia-Upjohn Diagnostic, Uppsala, Sweden) with rat insulin standard (Novo Research Institute, Bagsvaerd, Denmark).

### 2.11. Statistical Analyses

All data are expressed as the mean ± SD. Mean comparisons among multiple groups were conducted by one-way analysis of variance (ANOVA) using SPSS 14.0 software. Comparisons of the means between two groups were conducted using randomized controlled *t*-tests. A *p* value < 0.05 was considered statistically significant.

## 3. Results

### 3.1. HUA and Ang II Cooperate to Increase Endothelial Injury

**Figure 1:**
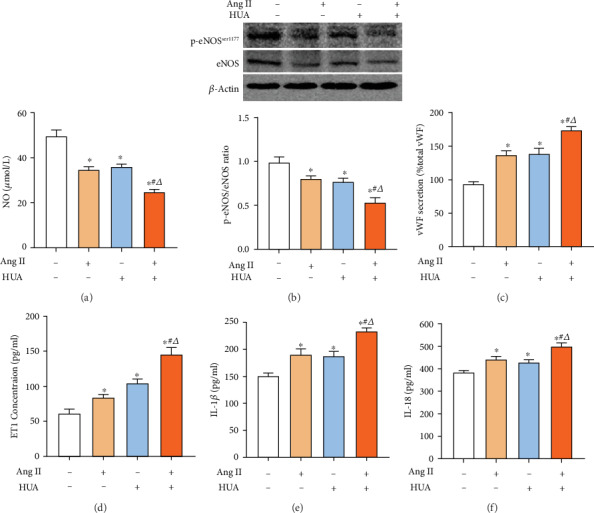
UA and Ang II induce endothelial injury. (a) The NO level of the supernatant was assayed by NO reduction. HUVECs were treated with 600 *μ*mol/L UA (HUA), 10^−7^ mol/L Ang II (Ang II), or 600 *μ*mol/L UA together with 10^−7^ mol/L Ang II (HUA+Ang II) to reduce NO production after 24 h of stimulation; ^∗^*p* < 0.05 compared to the normal control. The NO level in the HUA+Ang II group was significantly lower than that in the HUA group or the Ang II group; ^#^*p* < 0.05 compared to the Ang II group; ^△^*p* < 0.05 compared to the HUA group. (b) Western blotting was used to detect eNOS expression levels in HUVECs. The HUA, Ang II, and HUA+Ang II groups showed decreased phosphorated eNOS-ser1177 protein expression levels in HUVECs after 24 h of stimulation; ^∗^*p* < 0.05 compared to the normal control group. The phosphorated eNOS-ser1177 protein expression level in the HUA+Ang II group was significantly lower than that in the HUA group or the Ang II group; ^#^*p* < 0.05 compared to the Ang II group; ^△^*p* < 0.05 compared to the HUA group. (c–f) The vWF, ET1, IL-1*β*, and IL-18 concentration of the supernatant was assayed. HUA and Ang II increased vWF, ET1, IL-1*β*, and IL-18 production after 24 h of stimulation; ^∗^*p* < 0.05 compared to the normal control. The vWF, ET1, IL-1*β*, and IL-18 in the HUA+Ang II group were remarkably higher than those in the HUA group or the Ang II group; ^#^*p* < 0.05 compared to the Ang II group; ^△^*p* < 0.05 compared to the HUA group.

To confirm that HUA and Ang II cause endothelial injury, we measured NO in the cell culture medium after HUVECs were treated with 600 *μ*mol/L or 10^−7^ mol/L Ang II for 24 h. Compared to NO levels in the normal control, the NO level was significantly decreased in the HUA and Ang II groups (*p* < 0.05, Figure 1(a)). Furthermore, NO was remarkably lower in the HUA+Ang II group than that in the HUA and Ang II groups (*p* < 0.05, Figure 1(a)). Additionally, the phosphorated eNOS-ser1177 protein level in the HUA and Ang II groups was significantly downregulated compared to that in the control group, and eNOS levels were notably lower in the HUA+Ang II group than those in the HUA and Ang II groups (*p* < 0.05, Figure 1(b)), consistent with the significantly lower NO level in the HUA+Ang II group. Also, we assayed the endothelial cell injury marker vWF and ET1 concentration in the cell supplement; data showed vWF and ET1 in the HUA and Ang II groups were significantly increased when compared with normal control; particularly, they were notably higher in the HUA+Ang II group than those in the HUA and Ang II groups (Figures 1(c) and 1(d)). Previous study determinates HUA could mediate endothelial cell inflammation [10]; thus, we measured inflammatory mediators IL-1*β* and IL-18 concentration in the cell supplement; data showed IL-1*β* and IL-18 in the HUA and Ang II groups were significantly increased when compared with normal control; particularly, they were notably higher in the HUA+Ang II group than those in the HUA and Ang II groups (Figures 1(e) and 1(f)). Taken together, our data suggest that HUA effectively impairs endothelial cells and acts additively with Ang II.

### 3.2. HUA and Ang II Promote ROS Production via a Cooperative Affect

**Figure 2:**
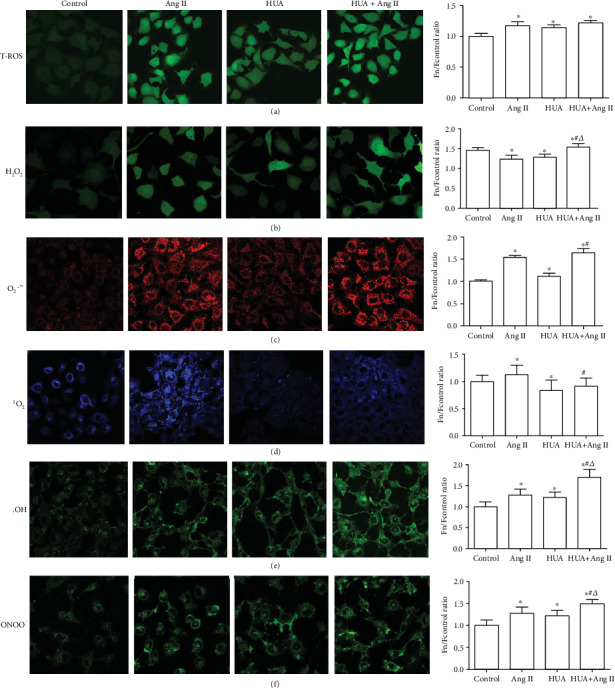
Total ROS production and ROS component generation in HUVECs induced by HUA or Ang II. HUVECs were cultured in confocal dishes and treated with 600 *μ*mol/L UA (HUA) or 10^−7^ mol/L Ang II (Ang II) for 24 h. Then, cells were stained using ROS probes. (a) Compared to the control group, the HUA, Ang II, and HUA+Ang II groups generated more total intracellular ROS (^∗^*p* < 0.05), and no difference was observed between the HUA+Ang II group and the HUA or Ang II group (*p* > 0.05). (b) The HUA, Ang II, and HUA+Ang II groups showed remarkably increased H_2_O_2_ production compared to the control group (^∗^*p* < 0.05), and HUA+Ang II treatment increased H_2_O_2_ generation compared to individual treatment with HUA or Ang II (^#^*p* < 0.05 compared to the HUA group; ^△^*p* < 0.05 compared to the Ang II group). (c) The HUA, Ang II, and HUA+Ang II groups showed remarkably higher O_2_^·-^ production than the control group (^∗^*p* < 0.05), and HUA+Ang II treatment increased O_2_^·-^ generation compared to individual treatment with HUA or Ang II (^#^*p* < 0.05 compared to the HUA group). (d) The ^1^O_2_ level was increased in the Ang II group (^∗^*p* < 0.05) and decreased in the HUA group compared to that in the control group (^∗^*p* < 0.05). The ^1^O_2_ level was lower in the HUA+Ang II group than that in the Ang II group (^#^*p* < 0.05); (e) the ^·^OH level was higher in the HUA, Ang II, and HUA+Ang II groups than that in the control group (^∗^*p* < 0.05) and was significantly higher in the HUA+Ang II group than that in the HUA and Ang II groups (^#^*p* < 0.01 compared to the HUA group; ^△^*p* < 0.01 compared to the Ang II group). (f) The ONOO^−^ level was higher in the HUA, Ang II, and HUA+Ang II groups than that in the control group (^∗^*p* < 0.05) and was significantly higher in the HUA+Ang II group compared to that in the HUA or Ang II group (^#^*p* < 0.01 compared to the HUA group; ^△^*p* < 0.01 compared to the Ang II group).

ROS are essential for an organism, but the constant accumulation of ROS can damage intracellular proteins, lipids, the nucleus, and mitochondrial DNA. The four major cellular ROS components are O_2_^·-^, ^·^OH, ^1^O_2_, and H_2_O_2_, all of which can be interconverted [15–19]. ONOO^−^ is not a cellular ROS component but is the reaction product of NO and O_2_^·−^. We measured the levels of total intracellular ROS and its components using different probes via confocal microscopy (Figure 2). Compared to the levels in the control group, the total ROS, H_2_O_2_, O_2_^·-^, ^·^OH, and ONOO^−^ levels in the HUA, Ang II, and HUA+Ang II groups were increased (*p* < 0.05). No difference was observed in the total ROS level between the HUA+Ang II group and the HUA or Ang II group (*p* > 0.05). However, the H_2_O_2_ and ^·^OH levels in the HUA+Ang II group were significantly higher than those in the HUA or Ang II groups (*p* < 0.05 and *p* < 0.01, respectively). Additionally, the O_2_^·−^ level in the HUA+Ang II group was remarkably higher than that in the HUA group (*p* < 0.05) but similar to that in the Ang II group (*p* > 0.05). Compared to the level in the normal control group, the ^1^O_2_ level was decreased in the HUA group (*p* < 0.05) but increased in the Ang II group (*p* < 0.05). The ^1^O_2_ level in the HUA+Ang II group was lower than that in the Ang II group (*p* < 0.05), but no difference was observed between the ^1^O_2_ level in the HUA and HUA+Ang II groups (*p* > 0.05). Therefore, these data indicated that HUA and Ang II aggravated the production of total intracellular ROS and intracellular H_2_O_2_, O_2_^·-^, ^·^OH, and ONOO^−^ and promoted oxidative stress. However, UA was able to partially scavenge ^1^O_2_.

### 3.3. HUA and Ang II Promote NOX Activity

**Figure 3:**
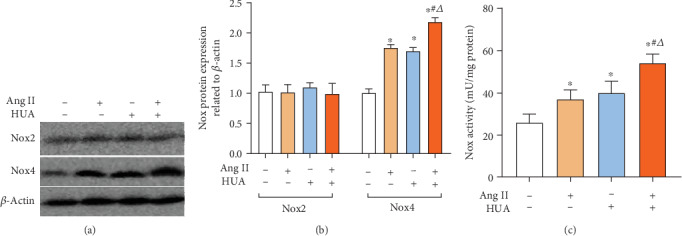
NOX expression in HUVECs induced by HUA or Ang II. (a, b) NOX2 and NOX4 protein expressions were assayed by Western blot. The HUA, Ang II, and HUA+Ang II groups showed increased NOX4 protein expression levels in HUVECs after 24 h of stimulation; ^∗^*p* < 0.05 compared to the normal control group, but there is no difference of NOX2 expression between each group. The NOX4 protein expression level in the HUA+Ang II group was significantly higher than that in the HUA group or the Ang II group; ^#^*p* < 0.05 compared to the Ang II group; ^△^*p* < 0.05 compared to the HUA group. (c) Also, NOX activity in HUA, Ang II, and HUA+Ang II groups increased remarkably when compared with normal control. Particularly, that was significantly higher in the HUA+Ang II group compared to that in the HUA or Ang II group; ^#^*p* < 0.05 compared to the Ang II group; ^△^*p* < 0.05 compared to the HUA group.

NADPH oxidase (NOX) is a well-characterized enzyme that generates high levels of superoxide and secondary oxidants [20]. Therefore, we detected two common NOXs, NOX2 and NOX4, in the endothelial cells which were induced injury by HUA or Ang II. Results show NOX4 protein and activity in the HUA or Ang II group were increased (Figure 3). Particularly, they were increased markedly in the HUA+Ang II group when compared to those in the HUA or Ang II group. However, there was no significant change of NOX2 protein level and activity among each group. It indicated that HUA could promote endothelial cell injury induced by Ang II via the NOX4 upregulation and producing excess ROS.

### 3.4. MS Model

**Figure 4:**
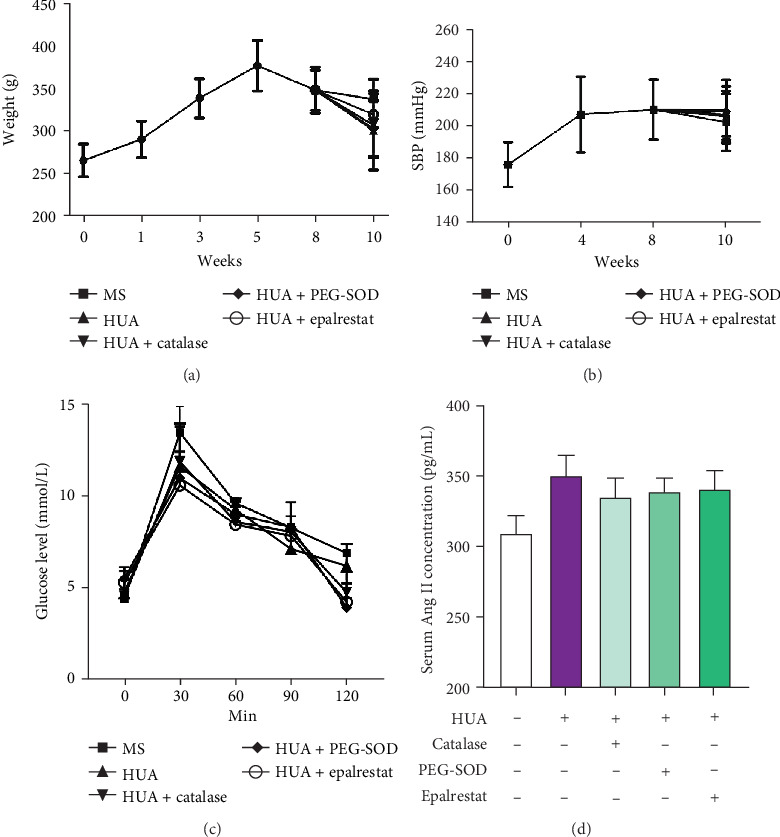
Body weight, systolic blood pressure, IGTT, and serum of Ang II of animals. (a) The body weight of animals in each group increased before 5 weeks of age and decreased after 8 weeks of age. No difference was observed among the groups (*p* > 0.05). (b) The systolic blood pressure increased to 200 mmHg at 4 weeks and maintained a steady state. No difference was observed among the groups (*p* > 0.05). (c) Intraperitoneal glucose tolerance of animals in each group has no differences, *p* > 0.05. (d) Serum Ang II level increased significantly in the HUA animal model, but catalase, PEG-SOD, and epalrestat did not decrease the Ang II concentration; there was no differences between the treatment groups and the HUA group, *p* > 0.05.

We constructed an MS model using SHR rats fed high-fat food. Figures 4(a) and 4(b) show that the weight and blood pressure of the animals increased with age. The blood pressure was stable in adult rats. These animals were treated with oxonic acid and UA to induce hyperuricemia and were administered PEG-SOD, catalase, and epalrestat.

After 2 weeks of treatment, body weight decreased, but no significant differences were found among the groups. Compared to baseline levels, the serum triglyceride (TG), low-density lipoprotein cholesterol (LDL-C), and blood glucose (BG) levels of each group were significantly increased (*p* < 0.05); however, no difference was found among the groups. The serum total cholesterol (TC) and high-density lipoprotein cholesterol (HDL-C) levels of each group increased after treatment but did not significantly differ from each other. Compared to the serum UA concentrations of the MS animals, the serum UA concentrations of the HUA+catalase, HUA+PGE-SOD, and HUA+epalrestat groups were remarkably increased (*p* < 0.05) (Tables 1 and 2). Meanwhile, we assayed intraperitoneal glucose tolerance and serum Ang II concentration in animals after treatment; results showed there was no differences of IGTT among those groups (Figure 4(c)); the serum Ang II level increased significantly in the HUA animal model, but catalase, PEG-SOD, and epalrestat did not decrease the Ang II concentration (Figure 4(d)).

### 3.5. ROS Scavenging Improves Endothelial Function in the MS Model

**Figure 5:**
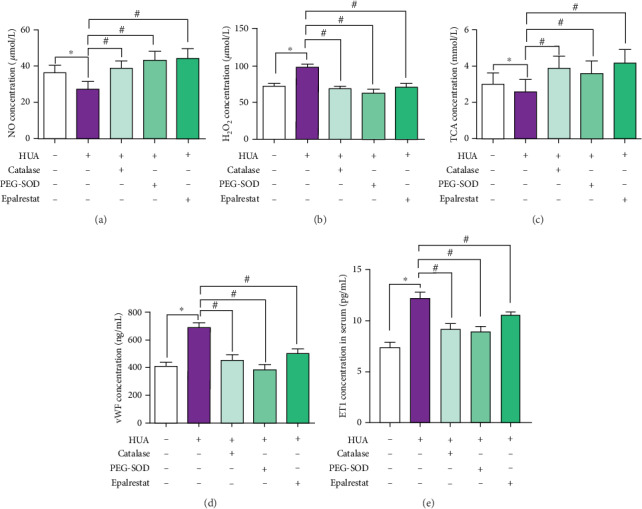
ROS scavenging improves endothelial function in the MS model. SHR rats were fed a high-fat and high-glucose diet and administered oxonic acid (250 mg kg^−1^ d^−1^) and UA (250 mg kg^−1^ d^−1^) via intraperitoneal injection to generate a model of MS associated with hyperuricemia. (a) In the HUA group, the serum NO level was significantly decreased compared to that in the SHR group (*p* < 0.05). After administration of catalase, PEG-SOD, or epalrestat, the serum NO level of HUA rats increased remarkably. (b) Compared to the H_2_O_2_ level in SHR rats, the H_2_O_2_ level in MS model rats was significantly increased (*p* < 0.05), and treatment with catalase, PEG-SOD, or epalrestat decreased H_2_O_2_ generation (*p* < 0.05). (c) Compared to the TAC of SHR rats, the TAC of MS model rats was significantly decreased (*p* < 0.05); treatment with and catalase, PEG-SOD, or epalrestat increased H_2_O_2_ generation (*p* < 0.05). (d) vWF and (e) ET1 are two markers of endothelial injury. In the HUA group, the serum vWF and ET1 concentration was significantly increased compared to that in the SHR group (*p* < 0.05). After administration of catalase, PEG-SOD, or epalrestat, the serum vWF and ET1 concentration in HUA rats increased remarkably (*p* < 0.05).

Compared to the levels in the MS animals, the serum NO level and TAC in the HUA group were significantly decreased, and the H_2_O_2_ level and concentration of the endothelial injury marker vWF and ET1 were increased (*p* < 0.05). Compared to the levels in the HUA-treated animals, the serum NO levels and TAC in the HUA+catalase, HUA+PEG-SOD, and HUA+epalrestat groups were significantly increased, and the H_2_O_2_ level and vWF and ET1 concentration were decreased (*p* < 0.05) (Figure 5).

## 4. Discussion

MS patients always exhibit hyperuricemia. One of the most often studied components of MS, Ang II, is also related to blood pressure regulation, endothelial damage, and vascular remolding. UA, which is generated in mammalian systems as an end product of purine metabolism, is the most abundant antioxidant in human plasma and possesses free radical scavenging properties. In humans and other higher primates, UA is the final compound of purine catabolism, but all other mammals convert UA to allantoin with the enzyme uricase, in which humans and other higher primates are deficient [21]. However, HUA is harmful to the health of humans. Markedly increased UA levels cause gout and nephrolithiasis [22] and are associated with an increased risk of developing cardiovascular disease (CVD), particularly hypertension, obesity/MS, and kidney disease [23–28]. Oxidative stress is the pivotal point in the process of endothelial dysfunction caused by hyperuricemia.

Researchers have demonstrated that endothelial dysfunction is associated with decreased NO production and increased ROS production [29]. Reduced NO release from endothelial cells is a marker of injury. In our study, Ang II and HUA caused a decrease in the NO level in the supernatant of HUVECs, and HUA+Ang II together further decreased NO release. The phosphorylated eNOS which is a synthetase of NO also showed similar trends. Additionally, studies have demonstrated that Ang-converting enzyme inhibition counteracts Ang II-mediated endothelial cell dysfunction [30]. Our in vitro data also showed endothelial cell injury marker vWF and ET1 markedly increased by HUA+Ang II. Therefore, we believe that Ang II and HUA act additively to cause endothelial cell injury. Besides those, we also found uric acid and Ang II not only induced IL-1beta and IL-18 secretion of endothelial cell individually, similarly with other reports [31, 32], but also UA can aggravate those inflammatory mediator secretions that are induced by Ang II. The mechanism maybe uric acid and Ang II triggers the activation of inflammasome such as NLRP3 [32, 33].

Evidence has shown that the production of oxygen-free radicals mediated by hyperuricemia is an important initiating factor of endothelial dysfunction [34]. The four major cellular ROS components are O_2_^·-^, ^·^OH, ^1^O_2_, and H_2_O_2_, all of which can be interconverted [15–19] and can damage cells. Oxidation-reduction reactions initially result in the production of O_2_^·-^, which can be converted into H_2_O_2_ and then converted into other ROS components; therefore, O_2_^·-^ and H_2_O_2_ are the most important ROS components [35].

Our study showed that Ang II increased O_2_^·-^, ^1^O_2_, H_2_O_2_, ^·^OH, and ONOO^−^ levels in HUVECs. Additionally, treatment with 600 *μ*mol/L UA increased O_2_^·-^, ^·^OH, and H_2_O_2_ levels but decreased the ^1^O_2_ level, which was also confirmed by a previous study [7]. O_2_^·-^ is converted to H_2_O_2_ by SOD. Our results indicated that HUA is unable to inhibit the release of O_2_^·-^ but promotes the production of O_2_^·-^, consistent with other reports [36]. Furthermore, we also assayed the ONOO^−^ level, which is associated with the development of endothelial dysfunction [37, 38]. Excessive levels of O_2_^·-^ reacted with NO to produce ONOO^−^. When cells were stimulated by Ang II combined with HUA, O_2_^·-^, H_2_O_2_, ^·^OH, and ONOO^−^ levels increased, but the ^1^O_2_ level decreased. Taken together, our results suggest that HUA increases total ROS production but scavenges ^1^O_2_.

To clarify the mechanism through which HUA and Ang II cause damage, we constructed an MS rat model with hyperuricemia. The model consisted of SHR rats fed high-fat and high-glucose chow for 8 weeks. The phenotype of the model included most of the features observed during the course of the natural human disease of MS [39]. The blood pressure of these animals began to increase at 5-6 weeks and continued to increase with age, with the systolic pressure in adult animals reaching 180-200 mmHg [40] [41]. During administration of the high-fat and high-glucose diet, the serum TG, LDL-C, and BG levels gradually increased, the abdominal fat thickened, and body weight increased rapidly. However, upon hyperglycemia aggravation, the increase in body weight slowed, and a decrease in body weight was even observed (see Figure 3). Also, we observed the renal function of MS rats declined with higher Scr and BUN when compared with the baseline data. Particularly, the renal function of HUA animals was damaged much more when compared with that of MS rats. The above data indicated Ang II and HUA aggravated the kidney damage. These phenomena can also be observed in MS patients [42].

The serum NO level and TAC were lower in the HUA group than those in the MS model, and the concentration of the endothelial injury marker vWF and H_2_O_2_ level was remarkably higher, indicating that endothelial injury occurred in the MS model, and hyperuricemia exacerbated the injury.

In the cell experiment, H_2_O_2_ and O_2_^·-^ were the key ROS and played an important role in the endothelial injury process. Therefore, we used catalase to clear H_2_O_2_ and PEG-SOD to clear O_2_^·-^ in the animal study. Our previous data demonstrated that HUA promotes an increase in both the expression and activity of AR in HUVECs [7, 8]. The AR inhibitor epalrestat decreased ROS production induced by HUA in HUVECs. AR participates in the processes of ischemia/reperfusion injury, diabetic neuropathy, and vascular damage and is also closely related to ROS production [43, 44]. Researchers have found that activation of AR increases the NADH/NAD^+^ ratio and decreases the NADPH/NADP^+^ ratio, which can accelerate ROS production and decrease NO, leading to endothelial injury [45]. We also discovered that NOX4, not NOX2, upregulated significantly in the HUA+Ang II treatment, and NOX activity increased as well. Therefore, we used the AR inhibitor epalrestat to treat animals. After administration of catalase, PEG-SOD, or epalrestat, the serum NO level and TAC increased remarkably, and the vWF concentration and H_2_O_2_ level decreased. These results indicated that removing H_2_O_2_ and O_2_^·-^ to block ROS production could relieve endothelial injury, further demonstrating that HUA and Ang II induce oxidative stress damage in endothelial cells and that AR plays a role in this process. Yet, we found three drugs did not provide benefit on the renal function, especially the BUN level of animals increased more than that of MS/UA animals. The probable cause could be that drugs were overdosed in the duration and animals were treated only 2 weeks. Thus, in the further study, we need to decrease the drug dosage and prolong the treatment period, then observe the renoprotection effect.

Taken together, these results suggest a new clinical target for the prevention and therapy of MS.

## 5. Conclusion

Hyperuricemia combined with MS can aggravate the endothelial injury caused by Ang II. Blocking the AR pathway or scavenging O_2_^·-^ and H_2_O_2_ may protect endothelial function. These actions will be a theoretical basis for the prevention and treatment of endothelial injury in patients with MS and hyperuricemia.

## Figures and Tables

**Table 1:** Changes of serum lipid of animals.

Groups	Number	TG (mmol/L)	TC (mmol/L)	LDL-C (mmol/L)	HDL-C (mmol/L)
Baseline	30	0.54 ± 0.15	1.55 ± 0.30	0.20 ± 0.10	1.51 ± 0.20
MS	6	2.88 ± 0.15^∗^	1.79 ± 0.12	0.31 ± 0.11^∗^	1.89 ± 0.19
HUA	6	2.80 ± 0.16^∗^	1.85 ± 0.14	0.41 ± 0.10^∗^	1.96 ± 0.13
HUA+catalase	6	2.66 ± 0.14^∗^	1.81 ± 0.19	0.4 ± 0.14^∗^	1.85 ± 0.11
HUA+PEG-SOD	6	2.74 ± 0.15^∗^	1.78 ± 0.17	0.41 ± 0.13^∗^	1.81 ± 0.18
HUA+epalrestat	6	2.78 ± 0.17^∗^	1.86 ± 0.13	0.43 ± 0.13^∗^	1.91 ± 0.12

^∗^ *p* < 0.05, compared with baseline.

**Table 2:** Uric acid, renal function, and blood glucose.

Groups	Number	UA (*μ*mol/L)	BG (mmol/L)	Scr (*μ*mol/L)	Bun (mmol/L)
Baseline	30	72.4 ± 8.98	4.80 ± 0.30	23.7 ± 2.72	7.45 ± 0.20
MS	6	66.0 ± 10.6	8.68 ± 1.12^∗^	47.8 ± 2.89^∗^	9.04 ± 1.09^∗^
HUA	6	101.1 ± 12.8^#^	8.61 ± 1.41^∗^	100.2 ± 11.2^∗^^#^	11.4 ± 1.33^∗^^#^
HUA+catalase	6	112.4 ± 11.8^#^	8.05 ± 1.53^∗^	137.7 ± 12.7^∗^^#^	12.6 ± 1.16^∗^^#^
HUA+PEG-SOD	6	98.8 ± 12.7^#^	8.80 ± 1.30^∗^	118.6 ± 10.2^∗^^#^	19.6 ± 1.28^∗^^#^
HUA+epalrestat	6	99.5 ± 10.7^#^	8.88 ± 1.24^∗^	141.1 ± 11.1^∗^^#^	19.5 ± 1.41^∗^^#^

^∗^ *p* < 0.05, compared with baseline. ^#^*p* < 0.05, compared with MS.

## Data Availability

The data that support the findings of this study are available from the corresponding author, WD, upon reasonable request.
